# Left atrial appendage occlusion versus antithrombotic therapy in nonvalvular AF with ASCVD and prior embolism

**DOI:** 10.1016/j.isci.2026.118036

**Published:** 2026-10-28

**Authors:** Chenlin Lu, Si Sun, Hao Liu, Lin Chen, Qingtao Ni, Yin Yuan, Jingcheng Chen, Chuanmeng Zhang, Zhongbao Ruan, Xiangwei Ding

**Affiliations:** 1 Department of Clinical Research Center, The Affiliated Taizhou People’s Hospital of Nanjing Medical University, Taizhou School of Clinical Medicine, Nanjing Medical University, Taizhou 225300, Jiangsu, China; 2 Department of Cardiology, The Affiliated Taizhou People’s Hospital of Nanjing Medical University, Taizhou School of Clinical Medicine, Nanjing Medical University, Taizhou 225300, Jiangsu, China; 3 Department of Cardiology, The Second People’s Hospital of Changzhou, The Third Affiliated Hospital of Nanjing Medical University, Changzhou 213000, Jiangsu, China; 4 Department of Cardiology, Suzhou Ninth Hospital Affiliated to Soochow University, Suzhou 215200, Jiangsu, China

**Keywords:** non-valvular atrial fibrillation, left atrial appendage occlusion, oral anticoagulation, antiplatelet therapy, recurrent embolism

## Abstract

Managing nonvalvular atrial fibrillation (NVAF) in patients with atherosclerotic cardiovascular disease (ASCVD) and prior embolism is challenging. This retrospective study evaluated long-term outcomes of left atrial appendage occlusion (LAAO) compared with oral anticoagulation (OAC) and antiplatelet therapy (APT) in primary (*n* = 647) and external validation (*n* = 570) cohorts. The primary composite outcome included recurrent ischemic stroke or systemic embolism, major bleeding, and all-cause mortality. After inverse probability of treatment weighting (IPTW), risks of the primary outcome, major bleeding, and mortality for LAAO were comparable with or lower than those of OAC and APT. For recurrent embolism, competing-risk analyses showed both OAC (SHR = 3.578, *p* = 0.003) and APT (SHR = 2.494, *p* = 0.040) carried a higher risk than LAAO. Results were consistent in the external validation cohort. These findings suggest LAAO may be a reasonable option for this high-risk population, offering a lower risk of recurrent embolism and comparable risk of major bleeding relative to pharmacological therapies.

## Highlights

• LAAO versus OAC and APT in NVAF patients with ASCVD and prior embolism
• LAAO was associated with lower recurrent embolism risk than either OAC or APT
• LAAO showed no excess major bleeding or mortality versus OAC or APT
• LAAO may be a reasonable option for this high-risk population

## Teaser

Health sciences

## Introduction

Atrial fibrillation (AF) is a major contributor to ischemic stroke.^1^^,^^2^ While oral anticoagulants (OACs) serve as the standard of care for stroke prevention, their clinical application is often limited by bleeding risks, adherence issues, and specific contraindications.^3^^,^^4^ Since approximately 90% of thrombi in non-valvular AF (NVAF) originate in the left atrial appendage (LAA), LAA occlusion (LAAO) has been established as a pivotal mechanical intervention for preventing cardioembolic stroke.^5^^,^^6^

Secondary prevention is particularly critical for AF patients with a prior history of embolism.^7^^,^^8^^,^^9^ Notably, a substantial proportion of these patients, particularly the elderly, present with comorbid atherosclerotic cardiovascular disease (ASCVD).^9^ The coexistence of AF and ASCVD poses a significant therapeutic dilemma: these patients face a dual risk of cardioembolism and atherothrombosis, leading to higher recurrence rates and poorer clinical outcomes. Clinically, this cohort is often characterized by advanced age, multiple comorbidities, and heightened concerns regarding both bleeding and medication adherence.^10^^,^^11^

Determining the optimal antithrombotic regimen for this complex population remains challenging. While OACs are the cornerstone for preventing AF-related embolism, antiplatelet therapy (APT) is fundamental for managing ASCVD.^11^^,^^12^ Theoretically, a combination of anticoagulant and antiplatelet drugs could offer more comprehensive protection when both conditions coexist. However, clinical evidence suggests that this combined strategy significantly increases the risk of major bleeding, especially intracranial hemorrhage, with uncertain net clinical benefit.^13^^,^^14^^,^^15^ Notably, recent randomized clinical trials demonstrated that in patients with ischemic stroke, concomitant AF, and systemic atherosclerosis, adding antiplatelet agents to anticoagulation failed to reduce recurrent ischemic events but increased the incidence of bleeding.^16^ This suggests the limitations of a purely pharmacological approach in balancing efficacy and safety for these high-risk patients.

LAAO offers a non-pharmacological alternative by mechanically excluding the primary source of cardioemboli. Pivotal trials such as PROTECT AF and PREVAIL established its non-inferiority to warfarin.^17^^,^^18^ More recently, the PRAGUE-17 trial and real-world registries extended these findings to high-risk patients, demonstrating comparable efficacy to direct oral anticoagulants (DOACs) but with a superior safety profile.^19^^,^^20^ Consequently, current international guidelines endorse LAAO as a recommended alternative for AF patients unsuitable for long-term OAC therapy.^8^^,^^21^

However, most evidence is derived from general AF populations. Real-world data directly comparing LAAO, OAC, and APT in high-risk patients with both prior embolism and comorbid ASCVD are scarce. We therefore conducted a retrospective cohort study to evaluate the long-term clinical outcomes of these three strategies. To assess the robustness of our results, we further validated these findings in an independent external validation cohort from two centers. Our objective was to identify the optimal secondary prevention approach for this challenging population.

## Results

### Baseline characteristics of the primary cohort

A total of 647 eligible patients were included in the primary cohort (Figure 1). The study population was divided into the LAAO group (*n* = 106), the OAC group (*n* = 318), and the APT group (*n* = 223). Within the OAC group, the vast majority of patients (*n* = 288, 90.6%) were prescribed DOACs. Among the remaining 30 patients (9.4%) treated with warfarin, the mean time in therapeutic range (TTR) during follow-up was 68.3% ± 11.2%, indicating adequate anticoagulation intensity. Regarding the specific subtypes of ASCVD, patients with atherothrombotic or lacunar stroke features were the most common (*n* = 401, 62.0%), followed by intracranial artery stenosis (*n* = 246, 38.0%), and carotid artery stenosis (*n* = 181, 28.0%). Additionally, 143 patients (22.1%) had comorbid ischemic heart disease, and 52 (8.0%) had peripheral artery disease.

**Figure 1:**
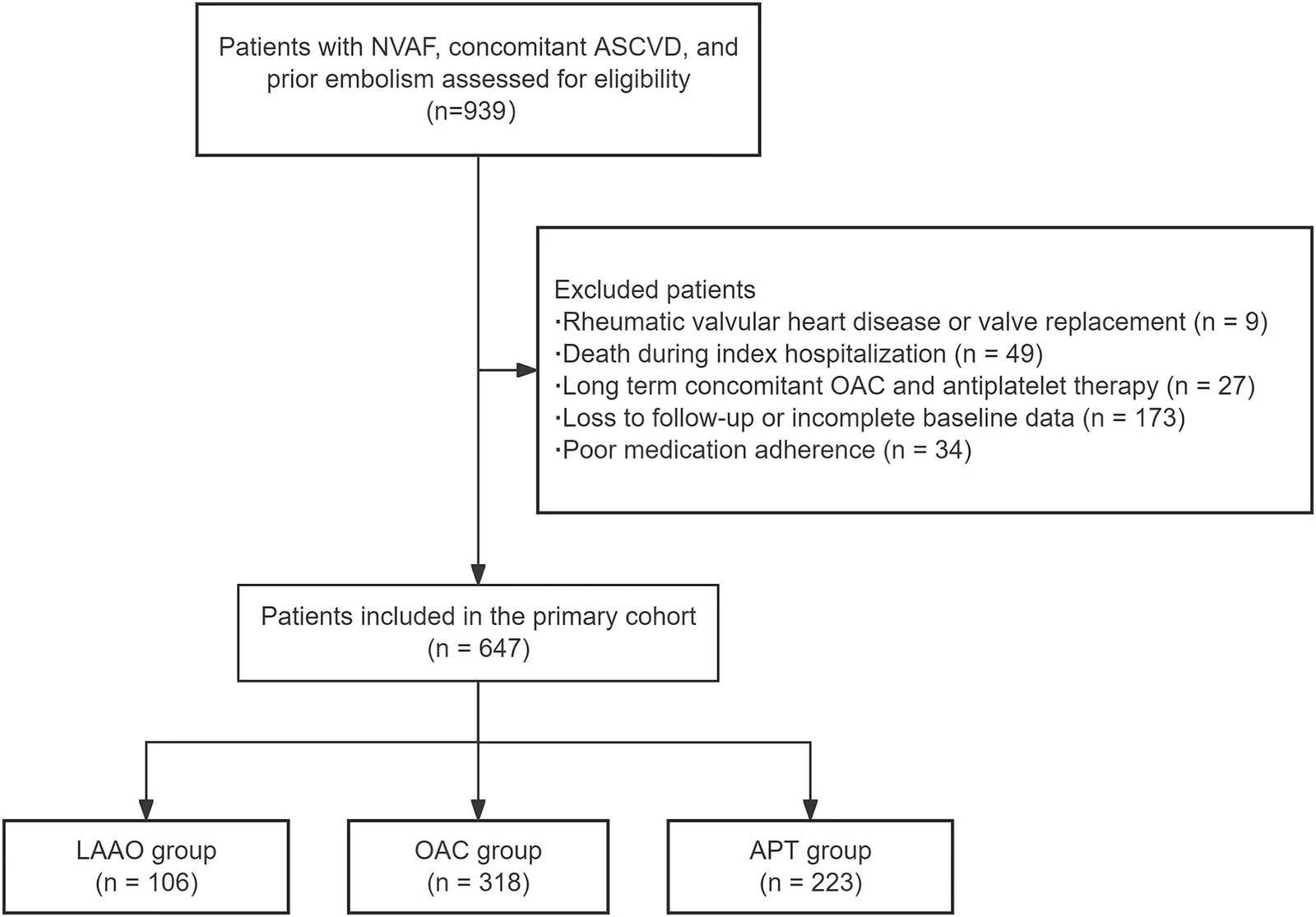
Flow chart of patient selection in the primary cohort NVAF, non-valvular atrial fibrillation; ASCVD, atherosclerotic cardiovascular disease; OAC, oral anticoagulation; APT, antiplatelet therapy; LAAO, left atrial appendage occlusion.

The overall study population was elderly. Patients in the LAAO group were younger (mean age 71.87 ± 9.39 years) compared with those in the OAC (76.11 ± 10.16 years) and APT groups (79.98 ± 8.34 years; *p* < 0.001). Sex distribution was comparable across groups (*p* = 0.087). The APT group had the highest median CHA_2_DS_2_-VASc score (5 [5–6]), compared with 5 [4–6] in both the LAAO and OAC groups (*p* < 0.001). The median HAS-BLED score was also highest in the APT group (3 [IQR 3–4]) versus 3 [IQR 2–3] in the other two groups (*p* < 0.001), indicating a higher overall bleeding risk in the APT group. Paroxysmal AF was most frequent in the LAAO group (26.4%), significantly higher than in the OAC (14.5%) and APT (19.3%) groups (*p* = 0.018). Regarding comorbidities, the prevalence of heart failure was highest in the OAC group (17.6%), compared with 7.5% in the LAAO group and 9.9% in the APT group (*p* = 0.005). Similarly, the prevalence of renal dysfunction was highest in the OAC group (20.8%), versus 11.3% in the LAAO group and 13.0% in the APT group (*p* = 0.017) (Table 1). After IPTW, baseline covariates were well balanced across the three groups, with all absolute standardized mean differences < 0.1 (Table 2).

**Table 1:** Baseline characteristics of patients in the primary cohort

Characteristic	LAAO group (*n* = 106)	OAC group (*n* = 318)	APT group (*n* = 223)	*p* value
Age, years, mean (SD)	71.87 ± 9.39	76.11 ± 10.16	79.98 ± 8.34	<0.001
Male, *n* (%)	65 (61.3%)	171 (53.8%)	108 (48.4%)	0.087
BMI, kg/m^2^, mean (SD)	24.62 ± 3.28	23.94 ± 3.69	23.04 ± 3.64	<0.001
Smoking, *n* (%)	26 (24.5%)	72 (22.6%)	40 (17.9%)	0.286
Alcoholism, *n* (%)	13 (12.3%)	25 (7.9%)	27 (12.1%)	0.192
Type of atrial fibrillation, *n* (%)				
Paroxysmal	28 (26.4%)	46 (14.5%)	43 (19.3%)	0.018
Persistent	78 (73.6%)	272 (85.5%)	180 (80.7%)	–
CHA_2_DS_2_-VASc score, median (IQR)	5 (4, 6)	5 (4, 6)	5 (5, 6)	<0.001
HAS-BLED score, median (IQR)	3 (2, 3)	3 (2, 3)	3 (3, 4)	<0.001
Hypertension, *n* (%)	68 (64.2%)	206 (64.8%)	155 (69.5%)	0.455
Diabetes, *n* (%)	34 (32.1%)	95 (29.9%)	64 (28.7%)	0.822
Coronary artery disease, *n* (%)	28 (26.4%)	78 (24.5%)	43 (19.3%)	0.240
Chronic heart failure, *n* (%)	8 (7.5%)	56 (17.6%)	22 (9.9%)	0.005
Myocardial infarction, *n* (%)	6 (5.7%)	26 (8.2%)	12 (5.4%)	0.391
Abnormal liver function, *n* (%)	6 (5.7%)	10 (3.1%)	12 (5.4%)	0.345
Abnormal renal function, *n* (%)	12 (11.3%)	66 (20.8%)	29 (13.0%)	0.017

BMI, body mass index; IQR, interquartile range.

**Table 2:** Baseline characteristics of the primary cohort after inverse probability of treatment weighting

Characteristic	LAAO group (weighted *n* = 100.93)	OAC group (weighted *n* = 317.53)	APT group (weighted *n* = 224.59)	*p* value	Absolute SMD
Age, years, mean (SD)	75.68 ± 9.02	76.69 ± 9.82	76.72 ± 9.55	0.649	0.074
Male, *n* (%)	55.2 (54.7%)	168.9 (53.2%)	121.4 (54.1%)	0.968	0.019
BMI, kg/m^2^, mean (SD)	23.80 ± 3.30	23.67 ± 3.69	23.72 ± 3.81	0.954	0.025
Smoking, *n* (%)	23.0 (22.8%)	67.5 (21.3%)	52.0 (23.2%)	0.894	0.03
Alcohol use, *n* (%)	8.8 (8.7%)	31.2 (9.8%)	23.2 (10.3%)	0.905	0.037
Type of atrial fibrillation: persistent, *n* (%)	82.3 (81.6%)	258.7 (81.5%)	184.2 (82.0%)	0.989	0.009
Hypertension, *n* (%)	67.0 (66.4%)	208.8 (65.8%)	149.9 (66.8%)	0.976	0.014
Diabetes, *n* (%)	28.1 (27.8%)	92.8 (29.2%)	67.1 (29.9%)	0.945	0.03
Coronary artery disease, *n* (%)	25.5 (25.3%)	75.2 (23.7%)	56.0 (25.0%)	0.936	0.025
Chronic heart failure, *n* (%)	11.8 (11.7%)	43.2 (13.6%)	28.5 (12.7%)	0.903	0.039
Myocardial infarction, *n* (%)	8.4 (8.3%)	22.7 (7.2%)	18.1 (8.1%)	0.931	0.028
Abnormal liver function, *n* (%)	4.5 (4.4%)	13.0 (4.1%)	10.1 (4.5%)	0.977	0.013
Abnormal renal function, *n* (%)	16.2 (16.0%)	51.2 (16.1%)	36.5 (16.3%)	0.998	0.005

LAAO, left atrial appendage occlusion; OAC, oral anticoagulation; APT, antiplatelet therapy; SD, standard deviation; BMI, body mass index; SMD, standardized mean difference.

Note: Absolute standardized mean differences were used to assess covariate balance across the three groups and an absolute SMD < 0.1 was considered to indicate acceptable balance.

### Primary and secondary outcomes in the primary cohort

The median follow-up durations were 4.08 years (IQR 2.90–6.11) in the LAAO group, 3.96 years (IQR 2.84–6.01) in the OAC group, and 4.51 years (IQR 3.20–5.81) in the APT group, with no significant difference across groups (*p* = 0.117).

The primary composite outcome occurred least frequently in the LAAO group (17.0%), significantly lower than in the OAC (37.7%) and APT (50.7%) groups (*p* < 0.001) (Table 3). Kaplan-Meier analysis confirmed significant disparities in cumulative event rates (overall log rank *p* < 0.001) (Figure 2). Pairwise comparisons demonstrated that the cumulative incidence in the LAAO group was significantly lower than in both the OAC (χ^2^ = 5.44, *p* = 0.020) and APT groups (χ^2^ = 12.84, *p* < 0.001), while the OAC group demonstrated a significantly lower incidence than the APT group (χ^2^ = 4.69, *p* = 0.030). In the multivariable Cox regression model, age was an independent predictor for the composite outcome (adjusted HR 1.024, 95% CI 1.006–1.042, *p* = 0.008). Compared with the LAAO group, antiplatelet therapy was associated with a significantly higher risk (adjusted HR 1.968, 95% CI 1.168–3.314, *p* = 0.011), whereas no significant difference was observed between LAAO and OAC for the composite outcome after adjustment (Table 4). In the IPTW-adjusted Cox analysis, the direction of the association remained similar, although the between-group differences were attenuated and were no longer statistically significant (OAC vs. LAAO: HR 1.217, 95% CI 0.688–2.152, *p* = 0.500; APT vs. LAAO: HR 1.516, 95% CI 0.851–2.702, *p* = 0.158) (Table 5).

**Table 3:** Unadjusted clinical outcomes and stroke severity in the primary cohort

	LAAO group	OAC group	APT group	*p* value
Composite outcome	18 (17.0%)	120 (37.7%)	113 (50.7%)	<0.001
Recurrent ischemic stroke/SE	8 (7.5%)	91 (28.6%)	56 (25.1%)	<0.001
Recurrent ischemic stroke	6 (5.7%)	77 (24.2%)	52 (23.3%)	<0.001
NIHSS score	3 (1.75, 6)	5 (3, 7.5)	10 (4, 14.75)	0.004
All-cause mortality	7 (6.6%)	40 (12.6%)	71 (31.8%)	<0.001
Major bleeding	5 (4.7%)	20 (6.3%)	22 (9.9%)	0.156

SE, systemic embolism.

**Figure 2:**
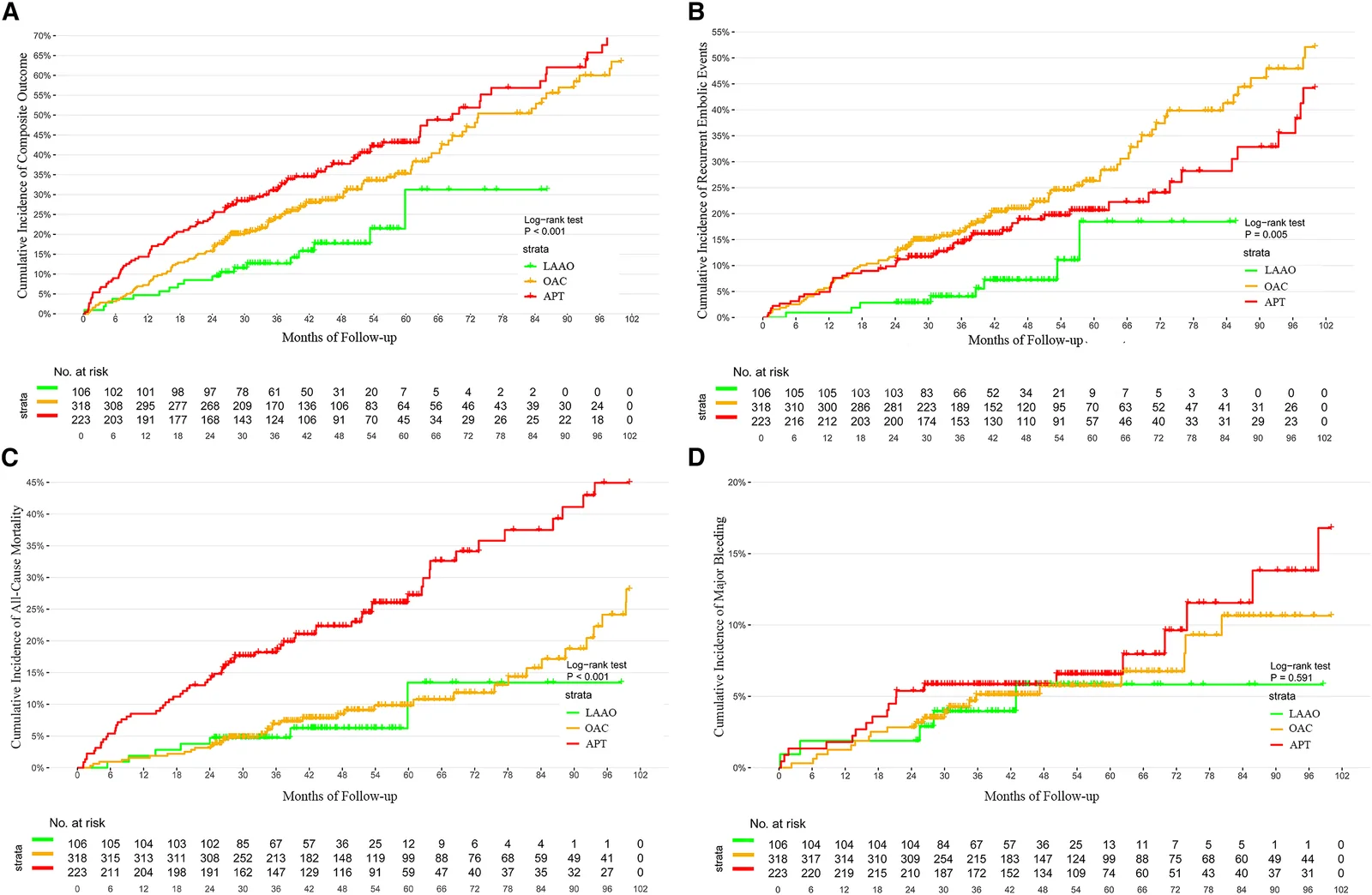
Kaplan-Meier curves of clinical outcomes during follow-up in the primary cohort (A) Cumulative incidence of the primary composite outcome. (B) Cumulative incidence of recurrent embolic events. (C) Cumulative incidence of all-cause mortality. (D) Cumulative incidence of major bleeding. LAAO, left atrial appendage occlusion; OAC, oral anticoagulation; APT, antiplatelet therapy.

**Table 4:** Univariate and multivariate Cox proportional hazards regression analyses for the primary composite outcome in the primary cohort

	Univariate analysis			Multivariate analysis		
	HR	95% CI	*p* value	HR	95% CI	*p* value
Gender	0.955	0.740–1.234	0.726	–	–	–
Age	1.033	1.018–1.048	<0.001	1.024	1.006–1.042	0.008
CHA_2_DS_2_-VASc score	1.14	1.039–1.252	0.006	1.012	0.897–1.142	0.845
HAS-BLED score	1.166	1.023–1.328	0.021	1.002	0.856–1.172	0.982
Hypertension	1.124	0.854–1.478	0.404	–	–	–
Diabetes	0.966	0.729–1.280	0.81	–	–	–
Coronary artery disease	0.816	0.599–1.112	0.198	–	–	–
Chronic heart failure	1.072	0.764–1.504	0.688	–	–	–
Myocardial infarction	1.365	0.880–2.117	0.165	–	–	–
Abnormal liver function	1.814	1.092–3.015	0.021	1.669	0.995–2.798	0.052
Abnormal renal function	1.385	1.021–1.878	0.035	1.219	0.889–1.670	0.219
Type of atrial fibrillation	1.273	0.885–1.830	0.193	–	–	–

**Treatment**						

LAAO	reference category			reference category		
OAC	1.818	1.103–2.997	0.019	1.583	0.953–2.630	0.076
APT	2.434	1.472–4.027	<0.001	1.968	1.168–3.314	0.011

HR, hazard ratio; CI, confidence interval; LAAO, left atrial appendage occlusion; OAC, oral anticoagulant; APT, antiplatelet therapy.

**Table 5:** IPTW-adjusted Cox proportional hazards analyses for clinical outcomes in the primary cohort

	Comparison	HR	95% CI	*p* value
Composite outcome	OAC vs. LAAO	1.217	0.688–2.152	0.500
	APT vs. LAAO	1.516	0.851–2.702	0.158
Recurrent embolism	OAC vs. LAAO	3.316	1.429–7.693	0.005
	APT vs. LAAO	2.437	1.019–5.826	0.045
Major bleeding	OAC vs. LAAO	1.187	0.426–3.305	0.743
	APT vs. LAAO	1.605	0.571–4.510	0.369
All-cause mortality	OAC vs. LAAO	0.753	0.288–1.966	0.562
	APT vs. LAAO	1.811	0.710–4.621	0.214

IPTW, inverse probability of treatment weighting; HR, hazard ratio; CI, confidence interval; LAAO, left atrial appendage occlusion; OAC, oral anticoagulation; APT, antiplatelet therapy.

The incidence of recurrent ischemic stroke or systemic embolism was 7.5% in the LAAO group, compared with 28.6% in the OAC group and 25.1% in the APT group (*p* < 0.001). Among the subset of patients who experienced recurrent ischemic stroke, the severity of neurological deficits at admission appeared lower in the LAAO group (median NIHSS 3 [IQR 1.75–6.00], *n* = 6) compared with the OAC (median 5 [IQR 3.00–7.50], *n* = 77) and APT groups (median 10 [IQR 4.00–14.75], *n* = 52; *p* = 0.004) (Table 3). Kaplan-Meier curves indicated significant differences among groups (overall log rank χ^2^ = 10.68, *p* = 0.005) (Figure 2). Pairwise comparisons showed that the LAAO group had a significantly lower cumulative risk than both the OAC (χ^2^ = 9.68, *p* = 0.002) and APT groups (χ^2^ = 5.01, *p* = 0.025). No significant difference was observed between the OAC and APT arms (χ^2^ = 2.22, *p* = 0.137). In multivariable analysis, using LAAO as the reference, both APT (adjusted HR 2.232, 95% CI 1.053–4.733, *p* = 0.036) and OAC therapy (adjusted HR 2.886, 95% CI 1.392–5.987, *p* = 0.004) were independently associated with an increased risk of recurrent embolism (Table 6). These associations remained significant in the IPTW-adjusted Cox analysis (OAC vs. LAAO: HR 3.316, 95% CI 1.429–7.693, *p* = 0.005; APT vs. LAAO: HR 2.437, 95% CI 1.019–5.826, *p* = 0.045) (Table 5). Furthermore, after accounting for the competing risk of death using the IPTW-adjusted Fine-Gray model, the results were materially unchanged (OAC vs. LAAO: SHR 3.578, 95% CI 1.540–8.312, *p* = 0.003; APT vs. LAAO: SHR 2.494, 95% CI 1.041–5.974, *p* = 0.040) (Table 7).

**Table 6:** Univariate and multivariate Cox proportional hazards regression analyses for recurrent embolism in the primary cohort

	Univariate analysis			Multivariate analysis		
	HR	95% CI	*p* value	HR	95% CI	*p* value
Gender	1.102	0.793–1.531	0.564	–	–	–
Age	1.012	0.994–1.030	0.192	–	–	–
CHA_2_DS_2_-VASc score	1.095	0.971–1.235	0.138	–	–	–
HAS-BLED score	1.054	0.890–1.248	0.542	–	–	–
Hypertension	1.343	0.935–1.929	0.111	–	–	–
Diabetes	1.252	0.885–1.770	0.205	–	–	–
Coronary artery disease	0.860	0.582–1.270	0.447	–	–	–
Chronic heart failure	0.659	0.400–1.085	0.101	–	–	–
Myocardial infarction	0.999	0.540–1.849	0.997	–	–	–
Abnormal liver function	1.035	0.484–2.212	0.930	–	–	–
Abnormal renal function	1.054	0.696–1.594	0.805	–	–	–
Type of atrial fibrillation	1.717	1.021–2.887	0.042	1.598	0.949–2.691	0.078

**Treatment**						

LAAO	reference category			reference category		
OAC	3.032	1.464–6.278	0.003	2.886	1.392–5.987	0.004
APT	2.319	1.095–4.911	0.028	2.232	1.053–4.733	0.036

HR, hazard ratio; CI, confidence interval; LAAO, left atrial appendage occlusion; OAC, oral anticoagulant; APT, antiplatelet therapy.

**Table 7:** IPTW-adjusted Fine-Gray competing risk analyses for recurrent embolism and major bleeding in the primary cohort

	Comparison	SHR	95% CI	*p* value
Recurrent ischemic stroke/SE	OAC vs. LAAO	3.578	1.540–8.312	0.003
	APT vs. LAAO	2.494	1.041–5.974	0.04
Major bleeding	OAC vs. LAAO	1.260	0.455–3.489	0.657
	APT vs. LAAO	1.629	0.580–4.573	0.354

IPTW, inverse probability of treatment weighting; SHR, subdistribution hazard ratio; CI, confidence interval; LAAO, left atrial appendage occlusion; OAC, oral anticoagulation; APT, antiplatelet therapy.

The all-cause mortality rate was 6.6% in the LAAO group, 12.6% in the OAC group, and 31.8% in the APT group (*p* < 0.001) (Table 3). Survival curves differed significantly among groups (overall log rank *p* < 0.001) (Figure 2). Pairwise comparisons indicated no significant difference between the LAAO and OAC groups (χ^2^ = 0.19, *p* = 0.660), but both groups had significantly lower mortality than the APT group (LAAO vs. APT: χ^2^ = 12.59, *p* < 0.001; OAC vs. APT: χ^2^ = 23.80, *p* < 0.001). In multivariable analysis, age (adjusted HR 1.054, 95% CI 1.026–1.082, *p* < 0.001) and hepatic dysfunction (adjusted HR 2.481, 95% CI 1.329–4.632, *p* = 0.004) were independent predictors of mortality. No significant association was found between treatment strategy and all-cause death after adjustment (Table 8). Similar findings were observed in the IPTW-adjusted Cox analysis, in which neither OAC (HR 0.753, 95% CI 0.288–1.966, *p* = 0.562) nor APT (HR 1.811, 95% CI 0.710–4.621, *p* = 0.214), compared with LAAO, was significantly associated with all-cause mortality (Table 5).

**Table 8:** Univariate and multivariate Cox proportional hazards regression analyses for all-cause mortality in the primary cohort

	Univariate analysis			Multivariate analysis		
	HR	95% CI	*p* value	HR	95% CI	*p* value
Gender	0.698	0.477–1.020	0.063	–	–	–
Age	1.073	1.048–1.099	<0.001	1.054	1.026–1.082	<0.001
CHA_2_DS_2_-VASc score	1.283	1.111–1.482	<0.001	1.05	0.874–1.261	0.603
HAS-BLED score	1.345	1.108–1.632	0.003	0.958	0.753–1.217	0.724
Hypertension	1.036	0.694–1.545	0.864	–	–	–
Diabetes	0.740	0.476–1.150	0.181	–	–	–
Coronary artery disease	0.685	0.422–1.114	0.128	–	–	–
Chronic heart failure	1.752	1.130–2.717	0.012	1.458	0.903–2.355	0.123
Myocardial infarction	1.689	0.926–3.079	0.087	–	–	–
Abnormal liver function	3.134	1.719–5.713	<0.001	2.481	1.329–4.632	0.004
Abnormal renal function	1.637	1.069–2.509	0.024	1.351	0.856–2.130	0.196
Type of atrial fibrillation	1.027	0.619–1.705	0.917	–	–	–

**Treatment**						

LAAO	reference category			reference category		
OAC	1.345	0.597–3.031	0.475	0.914	0.396–2.106	0.832
APT	3.554	1.621–7.790	0.002	2.258	0.998–5.111	0.051

HR, hazard ratio; CI, confidence interval; LAAO, left atrial appendage occlusion; OAC, oral anticoagulant; APT, antiplatelet therapy.

The incidence of major bleeding events did not differ significantly among the three groups (*p* = 0.156) (Table 3). Accordingly, Kaplan-Meier analysis showed no difference in cumulative incidence (overall log rank *p* = 0.591) (Figure 2), and none of the pairwise comparisons were significant. In the univariable Cox model, no variable, including treatment strategy, was significantly associated with major bleeding (Table 9). In the IPTW-adjusted Cox analysis, no significant difference in major bleeding was observed between LAAO and OAC (HR 1.187, 95% CI 0.426–3.305, *p* = 0.743) or between LAAO and APT (HR 1.605, 95% CI 0.571–4.510, *p* = 0.369) (Table 5). Similar results were obtained in the IPTW-adjusted Fine-Gray competing risk analysis with death treated as a competing event (OAC vs. LAAO: SHR 1.260, 95% CI 0.455–3.489, *p* = 0.657; APT vs. LAAO: SHR 1.629, 95% CI 0.580–4.573, *p* = 0.354) (Table 7).

**Table 9:** Univariate Cox proportional hazards regression analyses for major bleeding in the primary cohort

	Univariate analysis		
	HR	95% CI	*p* value
Gender	1.186	0.653–2.154	0.575
Age	1.017	0.985–1.050	0.312
CHA_2_DS_2_-VASc score	1.117	0.897–1.390	0.323
HAS-BLED score	1.286	0.950–1.741	0.103
Hypertension	2.084	1.002–4.337	0.050
Diabetes	1.133	0.600–2.136	0.701
Coronary artery disease	1.503	0.796–2.836	0.209
Chronic heart failure	0.810	0.340–1.929	0.634
Myocardial infarction	0.933	0.289–3.013	0.907
Abnormal liver function	1.036	0.251–4.280	0.961
Abnormal renal function	1.321	0.651–2.681	0.440
Type of atrial fibrillation	0.531	0.273–1.031	0.062

**Treatment**			

LAAO	reference category		
OAC	1.085	0.404–2.912	0.871
APT	1.452	0.539–3.915	0.461

HR, hazard ratio; CI, confidence interval; LAAO, left atrial appendage occlusion; OAC, oral anticoagulant; APT, antiplatelet therapy.

### External validation cohort

To assess the generalizability of our findings, we evaluated an independent external validation cohort of 570 eligible patients (LAAO, *n* = 112; OAC, *n* = 308; APT, *n* = 150). Baseline characteristics (Table S1) were balanced using the same IPTW approach as the primary analysis (all absolute SMDs <0.1; Table S3).

During a median follow-up of 4.32 (IQR 2.93–8.33) years, the clinical outcomes in the validation cohort were consistent with those of the primary cohort. The composite outcome occurred in 19.6% of patients in the LAAO group, compared with 30.2% in the OAC group and 40.7% in the APT group (*p* = 0.001) (Table S2). In the IPTW-adjusted Cox analysis using LAAO as the reference, APT was associated with a significantly higher risk of the composite outcome (HR 1.979, 95% CI 1.092–3.586, *p* = 0.024), whereas no significant difference was observed between the OAC and LAAO groups (HR 1.496, 95% CI 0.852–2.626, *p* = 0.160) (Table S4).

The incidence of recurrent embolism was 4.5% in the LAAO group, compared with 14.3% in the OAC group and 22.0% in the APT group (*p* < 0.001). Among the subset of patients who experienced recurrent ischemic stroke, the severity of neurological deficits at admission appeared lower in the LAAO group (median NIHSS 3 [IQR 1.50–5.50]) compared with the OAC (median 5 [IQR 3.00–7.75]) and APT groups (median 10 [IQR 7.00–13.50]; *p* < 0.001) (Table S2). In the IPTW-adjusted Cox analysis, both OAC (HR 3.141, 95% CI 1.159–8.510, *p* = 0.024) and APT (HR 5.347, 95% CI 1.932–14.792, *p* = 0.001) were associated with an increased risk of recurrent embolism compared with LAAO (Table S4). Furthermore, after accounting for the competing risk of death using the IPTW-adjusted Fine-Gray model, the results were materially unchanged (OAC vs. LAAO: SHR 3.093, 95% CI 1.141–8.386, *p* = 0.027; APT vs. LAAO: SHR 5.035, 95% CI 1.820–13.928, *p* = 0.002) (Table S5).

The all-cause mortality rate was 10.7% in the LAAO group, 14.0% in the OAC group, and 24.0% in the APT group (*p* = 0.005) (Table S2). In the IPTW-adjusted Cox analysis, APT was associated with a significantly higher risk of all-cause mortality when compared with LAAO (HR 2.391, 95% CI 1.131–5.054, *p* = 0.022), whereas no significant difference was observed between OAC and LAAO (HR 1.353, 95% CI 0.657–2.787, *p* = 0.413) (Table S4).

The incidence of major bleeding events did not differ significantly among the three groups (LAAO 5.4%, OAC 8.1%, APT 8.0%; *p* = 0.620). In the IPTW-adjusted Cox analysis, no significant difference in major bleeding was observed between LAAO and OAC (HR 1.133, 95% CI 0.315–4.072, *p* = 0.848) or between LAAO and APT (HR 0.896, 95% CI 0.231–3.471, *p* = 0.874). Similar results were obtained in the IPTW-adjusted Fine-Gray competing risk analysis with death treated as a competing event (OAC vs. LAAO: SHR 1.111, 95% CI 0.312–3.961, *p* = 0.871; APT vs. LAAO: SHR 0.839, 95% CI 0.219–3.211, *p* = 0.797) (Tables S2, S4, and S5).

## Discussion

This study evaluated three secondary prevention strategies in a complex population: patients with NVAF, concomitant ASCVD, and a history of embolism. To our knowledge, this is the first real-world retrospective cohort study to directly compare outcomes of LAAO, OAC, and APT in this specific group. Over a median follow-up of 4.2 years, although LAAO did not show a significant advantage for the primary composite outcome after IPTW adjustment, it was associated with a lower risk of recurrent embolic events compared with OAC and APT, without significant difference in major bleeding. Importantly, these findings were corroborated by an independent external validation cohort. These results suggest that LAAO may represent a reasonable alternative strategy for such high-risk patients with overlapping cardioembolic and atherosclerotic risks.

For AF patients with prior embolism, OAC is the standard care for secondary prevention.^22^ However, the relatively high recurrent embolism rate in our OAC group suggests that for patients with comorbid ASCVD, anticoagulation alone may be insufficient. The central debate in managing AF with concomitant ASCVD has consistently revolved around how to balance anticoagulation and antiplatelet therapy to optimize outcomes.^16^^,^^23^^,^^24^^,^^25^ Previous studies indicate that the mechanisms of embolic events in such patients are complex, involving not only cardioembolism from the LAA but also artery-to-artery embolism and *in situ* vessel occlusion secondary to unstable atherosclerotic plaques.^26^^,^^27^^,^^28^^,^^29^ While OAC is effective at inhibiting the coagulation factor-mediated cascade within the atria, it fails to adequately suppress the platelet-driven pathway dominant in atherosclerosis.^30^^,^^31^^,^^32^ This may explain the relatively high recurrence rate observed in our OAC group. In clinical practice, this residual ischemic risk has sometimes prompted consideration of intensified antithrombotic therapy, including the addition of antiplatelet agents. However, several studies suggest that this approach of intensifying systemic antithrombotic therapy may be accompanied by a significant increase in bleeding risk, thereby offsetting its potential vasculoprotective benefits.^16^^,^^25^^,^^33^ These findings indicate that relying solely on adjusting systemic antithrombotic medications makes it difficult to ideally balance efficacy and safety.

In contrast, LAAO provides an anatomy-based solution for this clinical dilemma. By mechanically excluding the LAA, LAAO eliminates over 90% of the cardiogenic thrombus sources.^34^^,^^35^ It replaces high-intensity systemic anticoagulation for AF-related stroke prevention with a physical barrier. This creates the condition for a de-escalation of the post-procedural antithrombotic regimen,^36^^,^^37^ potentially allowing clinicians to more safely employ antiplatelet regimens to focus on managing ASCVD risks. The reduced re-embolism rate and the non-increased rate of major bleeding in our LAAO group support this strategy. Among patients who experienced recurrent ischemic stroke, admission NIHSS scores were lower in the LAAO group. However, given the small number of recurrent stroke events in the LAAO group, this finding should be interpreted cautiously. This observation aligns with a plausible pathophysiological rationale: thrombi originating from the LAA are often large in volume and prone to causing occlusion of major vessels, leading to severe neurological deficits.^35^^,^^38^^,^^39^ In contrast, the residual risk after LAAO may stem more from *in situ* small vessel disease or tiny arterial emboli, events which cause relatively smaller areas of brain tissue damage. However, the lack of a significant between-group difference in major bleeding should be interpreted cautiously. Although previous studies have generally shown a lower long-term bleeding risk after LAAO, this benefit was not statistically evident in our cohort. Several factors may account for this finding. First, the absolute number of bleeding events was relatively small, particularly in the LAAO group, which may have limited statistical power. Second, LAAO is associated with peri-procedural bleeding risk, and patients still required intensified antithrombotic therapy during the early post-procedural period, potentially attenuating any later bleeding advantage. Third, our cohort consisted of patients with high baseline clinical risk and substantial comorbidity burden, which may have reduced the magnitude of any observable between-group difference in bleeding outcomes.

Conversely, the poor outcomes observed in the APT group serve as a warning. Relying solely on antiplatelet agents offers insufficient protection against recurrent events in this high-risk population. Focusing exclusively on vascular sclerosis while neglecting effective AF management is a hazardous strategy.

### Conclusion

In conclusion, our study suggests that in high-risk patients with NVAF, concomitant ASCVD, and prior embolism, LAAO may be reasonable alternative to conventional pharmacological therapy. Although LAAO did not show a significant advantage over OAC regarding the composite outcome, it was associated with a lower risk of recurrent ischemic stroke and systemic embolism compared with both pharmacological strategies, with no significant increase in major bleeding observed. These findings provide real-world evidence to guide personalized secondary stroke prevention strategies in this complex population. Prospective randomized controlled trials are warranted to further validate these findings.

### Limitations of the study

Several limitations of this retrospective cohort study merit consideration. Although we used multivariable adjustments and IPTW to balance measured baseline characteristics, and included an external validation cohort to enhance generalizability, the influence of potential unmeasured or residual confounding cannot be fully eliminated. For instance, patients opting for LAAO may have higher socioeconomic status or greater health literacy, both of which are factors often associated with better adherence and outcomes. Such differences may influence the observed treatment effect. Additionally, a small subset of patients in the LAAO group underwent concomitant catheter ablation. Catheter ablation itself may reduce AF burden and independently influence stroke risk, representing a potential confounding factor when evaluating the effect of LAAO alone.^40^ Furthermore, while we required strict medication adherence for inclusion, excluded patients who permanently switched between treatment groups before reaching an endpoint, and confirmed adequate TTR for warfarin users, antithrombotic therapy was still classified according to the initial treatment strategy during follow-up. Temporary treatment interruptions, dosage adjustments, or short-term changes in antithrombotic intensity may therefore not have been fully captured. Additionally, while we observed lower admission NIHSS scores among LAAO patients who experienced recurrent strokes, the absolute number of these events in the LAAO group was small (*n* = 6 in the primary cohort), and this finding should therefore be interpreted with caution. Finally, we did not stratify the analysis between DOACs and warfarin within the OAC group, nor did we examine the heterogeneity of outcomes based on specific ASCVD distributions. Future prospective randomized trials are imperative to definitively establish the optimal management strategy for this high-risk population.

## Resource availability

### Lead contact

Requests for further information and resources should be directed to and will be fulfilled by the lead contact, Xiangwei Ding (dingxw1208@126.com).

### Materials availability

This study did not generate new unique reagents.

### Data and code availability

The data for this study are available from the lead contact upon reasonable request. This paper does not report original code. Any additional information required to reanalyze the data reported in this paper is available from the lead contact upon request.

## Acknowledgments

The study was supported by the Research Program of Taizhou School of Clinical Medicine, 10.13039/501100007289Nanjing Medical University (TZKY20230209).

## Author contributions

C.L. performed the statistical analyses and drafted the manuscript. S.S., Y.Y., and Q.N. collected and verified the data for the primary cohort. H.L., L.C., and J.C. collected and verified the data for the external cohort. C.Z. provided statistical guidance. Z.R. and X.D. supervised the study, interpreted the data, and critically revised the manuscript. X.D. secured the funding. All authors have read and approved the final manuscript.

## Declaration of interests

The authors declare no competing interests.

## STAR★Methods

### Key resources table

**Table:** 

REAGENT or RESOURCE	SOURCE	IDENTIFIER
**Software and algorithms**		

SPSS Statistics 27.0	IBM	https://www.ibm.com/products/spss-statistics
R 4.5.2	The R Foundation	https://www.r-project.org/

### Experimental model and study participant details

#### Study population

This was a retrospective cohort study. We enrolled hospitalized patients with AF, comorbid ASCVD, and a documented history of ischemic stroke or systemic embolism. The primary cohort included patients from Taizhou People’s Hospital between January 2019 and June 2023. The external cohort comprised patients from The Second People’s Hospital of Changzhou and Suzhou Ninth Hospital Affiliated to Soochow University between January 2016 and December 2023. The study adhered to the principles of the Declaration of Helsinki. It was approved by the Ethics Committee of Taizhou People’s Hospital (Approval No: LSKY 2025–170–01). Owing to the retrospective design of the study, the requirement for written informed consent was waived. The baseline demographics, including age and sex, are summarized in Table 1 and Table S1. Regarding ethnicity, the entire study population was predominantly Han Chinese.

In both cohorts, patients were included if they met all of the following criteria: (1) age ≥18 years; (2) diagnosis of AF (paroxysmal, persistent, or permanent), confirmed by electrocardiography or Holter monitoring; (3) comorbid ASCVD, defined as a history of coronary artery disease (prior myocardial infarction, revascularization, or ≥50% stenosis on angiography), peripheral artery disease, or complex aortic plaque (≥4 mm thick or ulcerated); and (4) a documented history of ischemic stroke or systemic embolism verified by imaging. Patients were excluded if they had any of the following: (1) rheumatic valvular heart disease or a history of valve replacement; (2) long-term combined use of oral anticoagulation and antiplatelet therapy, excluding temporary antithrombotic regimens prescribed during the post-procedural transition period; (3) death during the index hospitalization or loss to follow-up; or (4) incomplete baseline clinical data or poor medication adherence (Figures 1 and S1).

### Method details

#### Procedures

Patients were stratified into three groups according to the treatment strategy prescribed at discharge. The LAAO group consisted of patients who underwent successful percutaneous left atrial appendage occlusion, defined as proper device positioning, absence of device-related thrombus, and no significant residual leak (leak width ≤5 mm). In this group, post-procedural antithrombotic regimens followed device instructions and 2019 AHA/ACC/HRS guidelines. In general, therapy consisted of a DOAC plus single antiplatelet therapy for the first 45 days, followed by dual antiplatelet therapy until 6 months, and single antiplatelet therapy thereafter. The OAC group comprised patients treated with long-term, standard-dose oral anticoagulation, including warfarin (target INR 2.0–3.0) or direct oral anticoagulants (DOACs: rivaroxaban, dabigatran, edoxaban, or apixaban). The APT group included patients who received neither LAAO nor OACs but were managed with single or dual antiplatelet therapy.

All included patients were required to meet a predefined criterion for “good medication adherence”, defined as adherence to the prescribed antithrombotic regimen for at least 80% of the follow-up period. Follow-up was conducted using electronic medical records, outpatient visits, and telephone interviews. Scheduled assessments occurred at 1, 3, 6, and 12 months after discharge, and every 6 months thereafter. At each interval, data were systematically collected regarding outcome events (time, type, and severity), current medication status, adherence, and any regimen adjustments. Follow-up commenced from the date when the assigned treatment was initiated after the embolic event: the LAAO procedure date for the LAAO group, and the OAC or APT therapy start date for the respective medication groups. A minimum follow-up of 2 years was required for all patients, continuing until the study end date (December 31, 2025) or death.

Patients who permanently crossed over from one treatment group to another before experiencing a clinical endpoint were excluded from the final analysis. For patients treated with warfarin in the OAC group, routine INR monitoring was performed and time in therapeutic range (TTR) was calculated.

#### Data collection and variable definitions

Baseline demographic data, AF type, history of ASCVD, and details of the embolic event were systematically recorded. We also documented comorbidities, including hypertension, diabetes mellitus, coronary artery disease, heart failure, and prior myocardial infarction. Key laboratory parameters, specifically renal and hepatic function were obtained.

Stroke and bleeding risk were quantified using the CHA_2_DS_2_-VASc and HAS-BLED scores, respectively. The CHA_2_DS_2_-VASc score was calculated according to established criteria: 2 points were assigned for age ≥75 years or a prior history of ischemic stroke/transient ischemic attack (TIA); 1 point each was assigned for age 65–74 years, female sex, congestive heart failure, hypertension, diabetes mellitus, or vascular disease (including myocardial infarction, peripheral artery disease, or aortic plaque). The HAS-BLED score, assessing bleeding risk, incorporated hypertension, abnormal renal/liver function, prior stroke, bleeding history, labile international normalized ratio (INR), age >65 years, and concomitant drug/alcohol use.

For patients who experienced a recurrent ischemic stroke, stroke severity at admission was assessed by neurologists using the National Institutes of Health Stroke Scale (NIHSS). This scale comprises 15 items evaluating consciousness, gaze, visual fields, facial palsy, and limb motor function, with total scores ranging from 0 to 42; higher scores indicate greater neurological deficit. Specific definitions were applied for organ dysfunction: hepatic dysfunction was defined as either a documented history of liver disease or an admission alanine aminotransferase/aspartate aminotransferase level ≥2 times the upper limit of normal; renal dysfunction was defined as an estimated glomerular filtration rate (eGFR) < 60 mL/min/1.73 m^2^. Data extraction was performed independently by two investigators, with any discrepancies resolved by a third reviewer.

#### Outcomes

The primary outcome was a composite of recurrent ischemic stroke, systemic embolism, major bleeding, and all-cause death. Secondary outcomes included the individual components of the primary composite, assessed separately.

Given the study cohort’s concomitant ASCVD, “systemic embolism” was defined as acute vascular occlusion outside the central nervous system, confirmed by imaging or surgical findings. This endpoint comprised events of either cardioembolic or atherothrombotic origin. Major bleeding was defined according to the criteria of the International Society on Thrombosis and Haemostasis (ISTH).^41^

### Quantification and statistical analysis

#### Statistical analysis

Continuous variables were tested for normality using the Shapiro-Wilk test, and presented as mean ± standard deviation (SD) or median (interquartile range, IQR), with intergroup differences evaluated by ANOVA or the Kruskal-Wallis H test, respectively. Categorical variables were described as frequency and percentage, compared using Pearson’s χ^2^ test or Fisher’s exact test. Survival curves were generated using the Kaplan-Meier method, and differences between groups were compared with the log rank test. Cox proportional hazards regression was used to evaluate the independent effect of treatment strategies on outcomes, adjusting for potential confounders arising from baseline imbalances. Variables with a *p* value <0.05 in univariable analysis were included in the multivariable Cox model for adjustment. Adjusted hazard ratios (HRs) with corresponding 95% confidence intervals (CIs) were calculated.

To further address baseline differences among the three groups, inverse probability of treatment weighting (IPTW) based on multinomial propensity scores was performed. For each patient, the probabilities of receiving LAAO, OAC, or APT were estimated using a multinomial logistic regression model including age, sex, BMI, smoking status, alcohol use, AF type, hypertension, diabetes mellitus, coronary artery disease, chronic heart failure, prior myocardial infarction, abnormal liver function, and abnormal renal function. Balance after weighting was evaluated using absolute standardized mean differences, and a value <0.10 was considered acceptable. Weighted Cox proportional hazards models were used for outcome analyses. Because death could preclude the occurrence of recurrent embolic events and major bleeding, Fine-Gray competing risk regression analyses after IPTW were also performed for these outcomes, with all-cause death treated as a competing event. Subdistribution hazard ratios (SHRs) and 95% CIs were reported.

Statistical analyses were performed using SPSS 27.0 and R 4.5.2. A two-sided *p* value <0.05 was considered statistically significant.
