# Supplementary material for: Left atrial appendage occlusion versus antithrombotic therapy in nonvalvular AF with ASCVD and prior embolism
Source: iScience. 2026 Oct 28;29(11):118036. doi: 10.1016/j.isci.2026.118036 (PMC13733314; doi:10.1016/j.isci.2026.118036)
Supplement: Supplementary file 1 [file mmc1.pdf]

iScience, Volume *29*

## Supplemental information

# Left atrial appendage occlusion versus antithrombotic therapy in nonvalvular AF with ASCVD and prior embolism

**Chenlin Lu, Si Sun, Hao Liu, Lin Chen, Qingtao Ni, Yin Yuan, Jingcheng Chen, Chuanmeng Zhang, Zhongbao Ruan, and Xiangwei Ding**

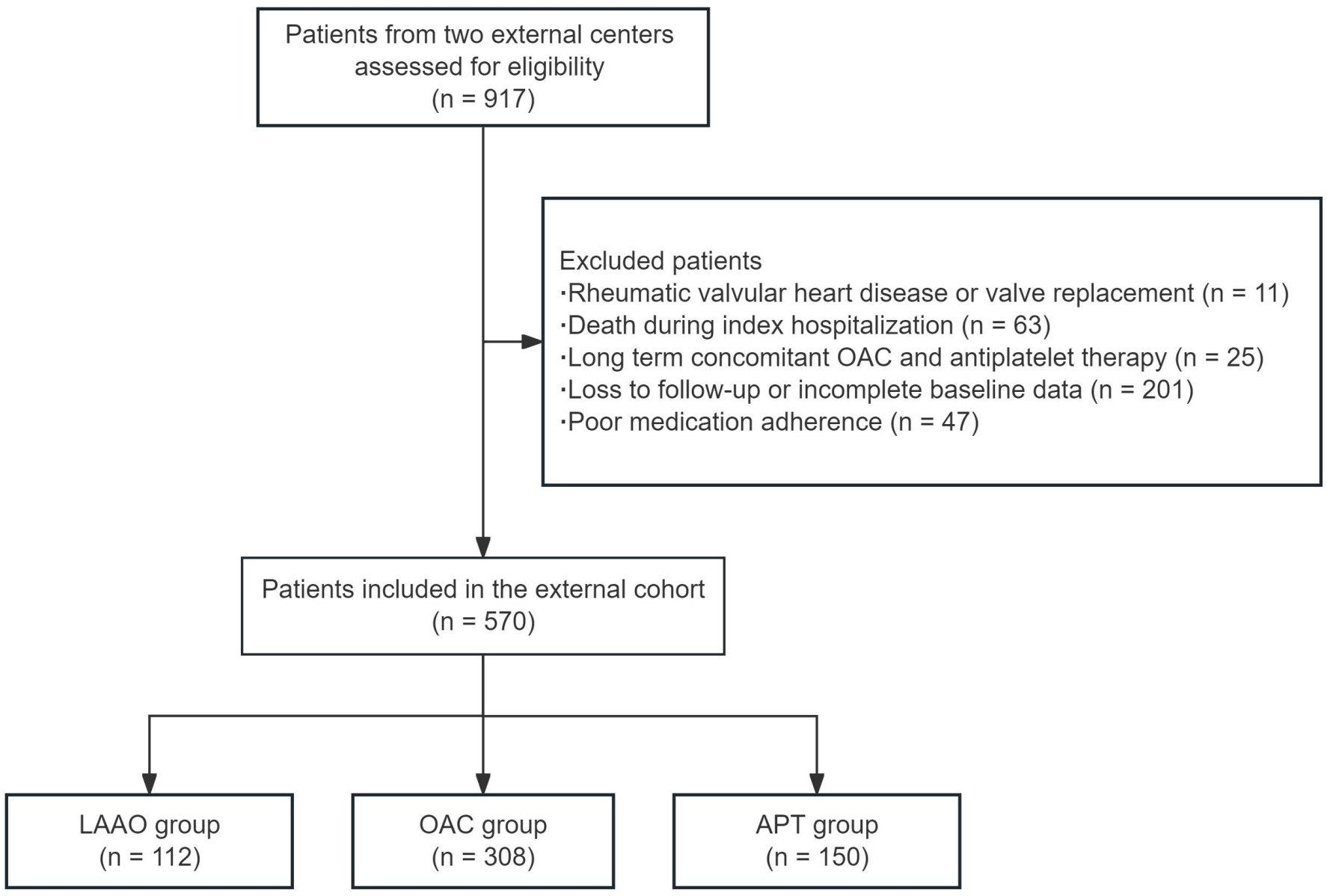


**Supplementary Figure S1. Flowchart of patient selection in the external cohort**

Abbreviations: OAC: oral anticoagulation; APT: antiplatelet therapy; LAAO: left atrial appendage occlusion

**Supplementary Table S1. Baseline characteristics of patients in the external validation cohort**

| Characteristic | LAAO group (n=112) | OAC group (n=308) | APT group (n=150) | *P* value |
|---|---|---|---|---|
| Age, y, mean (SD) | 71.56 ± 7.95 | 74.85 ± 8.19 | 76.93 ± 7.80 | <0.001 |
| Male, n (%) | 68 (60.7) | 181 (58.8) | 74 (49.3) | 0.101 |
| BMI, kg/m², mean (SD) | 24.04 ± 2.90 | 23.93 ± 3.55 | 23.75 ± 3.44 | 0.787 |
| Smoking, n (%) | 21 (18.8) | 52 (16.9) | 20 (13.3) | 0.464 |
| Alcoholism, n (%) | 15 (13.4) | 19 (6.2) | 11 (7.3) | 0.05 |
| Type of atrial fibrillation, n (%) | | | | <0.001 |
| Paroxysmal | 38 (33.9) | 39 (12.7) | 29 (19.3) | |
| Persistent | 74 (66.1) | 269 (87.3) | 121 (80.7) | |
| $CHA_2DS_2$-VASc score, median (IQR) | 5.00 (4.00, 6.00) | 5.00 (4.00, 6.00) | 5.00 (5.00, 6.00) | <0.001 |
| HAS-BLED score, median (IQR) | 3.00 (2.00, 3.00) | 2.00 (2.00, 3.00) | 3.00 (3.00, 4.00) | <0.001 |
| Hypertension, n (%) | 84 (75.0) | 221 (71.8) | 112 (74.7) | 0.713 |
| Diabetes, n (%) | 24 (21.4) | 70 (22.7) | 35 (23.3) | 0.934 |
| Coronary artery disease, n (%) | 27 (24.1) | 82 (26.6) | 33 (22.0) | 0.549 |
| Chronic heart failure, n (%) | 18 (16.1) | 56 (18.2) | 20 (13.3) | 0.419 |
| Myocardial infarction, n (%) | 5 (4.5) | 14 (4.5) | 7 (4.7) | 0.997 |
| Abnormal liver function, n (%) | 3 (2.7) | 7 (2.3) | 4 (2.7) | 0.954 |
| Abnormal renal function, n (%) | 7 (6.2) | 36 (11.7) | 17 (11.3) | 0.257 |

Abbreviations: BMI: body mass index; IQR: interquartile range.

**Supplementary Table S2. Unadjusted clinical outcomes and stroke severity in the external validation cohort**

| Outcome | LAAO group | OAC group | APT group | *P* value |
|---|---|---|---|---|
| Composite outcome | 22 (19.6%) | 93 (30.2%) | 61 (40.7%) | 0.001 |
| Recurrent ischemic stroke/SE | 5 (4.5%) | 44 (14.3%) | 33 (22.0%) | <0.001 |
| Recurrent ischemic stroke | 5 (4.5%) | 36 (11.7%) | 29 (19.3%) | 0.001 |
| NIHSS score | 3 (1.5, 5.5) | 5 (3, 7.75) | 10 (7, 13.5) | <0.001 |
| All-cause mortality | 12 (10.7%) | 43 (14.0%) | 36 (24.0%) | 0.005 |
| Major bleeding | 6 (5.4%) | 25 (8.1%) | 12 (8.0%) | 0.620 |

Abbreviations: SE: systemic embolism.

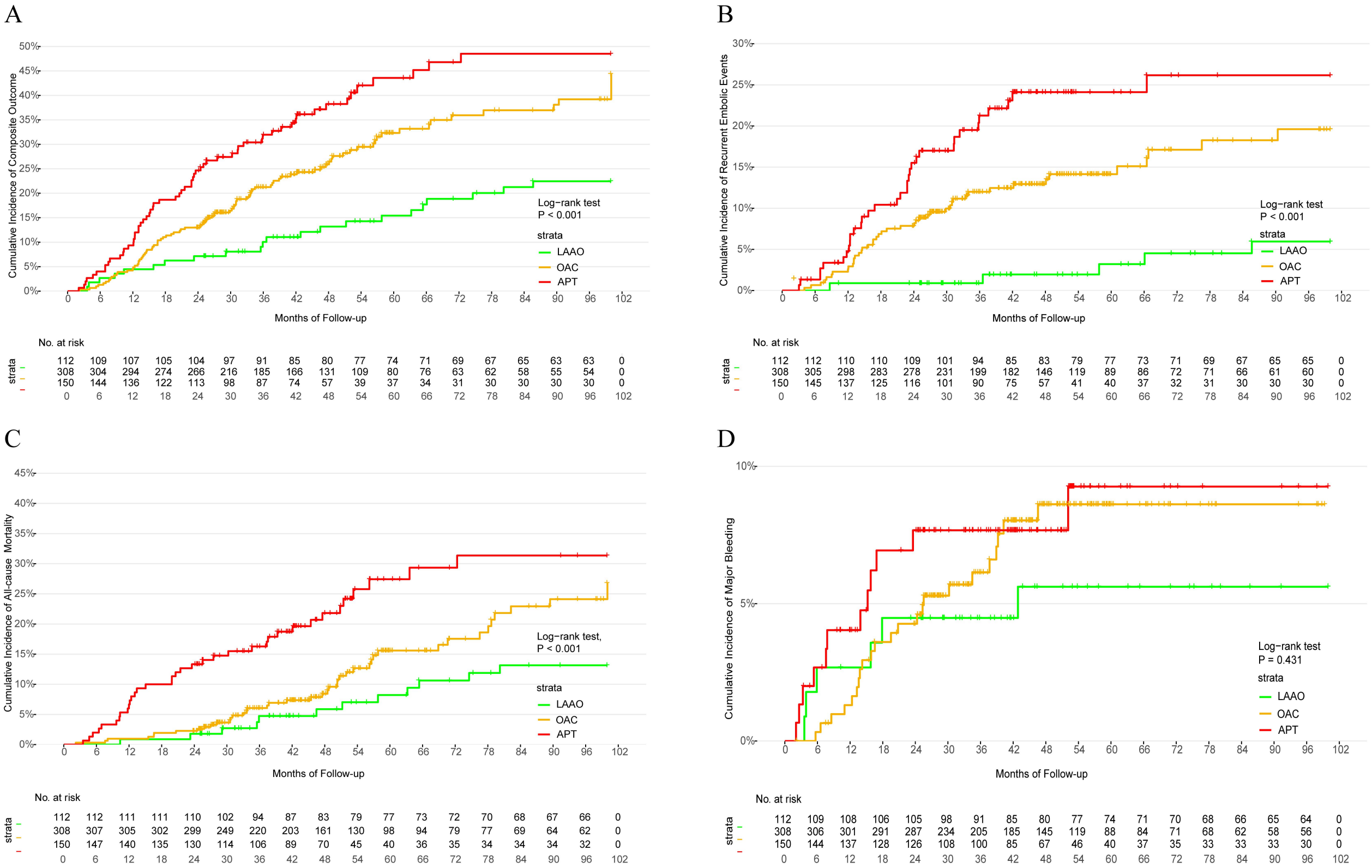


**Supplementary Figure S2. Kaplan-Meier curves of clinical outcomes during follow-up in the external validation cohort.** (A) Cumulative incidence of the primary composite outcome. (B) Cumulative incidence of recurrent embolic events. (C) Cumulative incidence of all-cause mortality. (D) Cumulative incidence of major bleeding.

Abbreviations: LAAO, left atrial appendage occlusion; OAC, oral anticoagulation; APT, antiplatelet therapy.

**Supplementary Table S3. Baseline characteristics of the external validation cohort after inverse probability of treatment weighting**

| Characteristic | LAAO group (weighted n=113.39) | OAC group (weighted n=307.94) | APT group (weighted n=150.32) | *P* value | Absolute SMD |
|---|---|---|---|---|---|
| Age, y, mean (SD) | 74.79 ± 8.36 | 74.73 ± 8.20 | 74.79 ± 8.15 | 0.997 | 0.008 |
| Male, n (%) | 68.5 (60.4) | 175.4 (57.0) | 87.4 (58.2) | 0.848 | 0.07 |
| BMI, kg/m², mean (SD) | 23.80 ± 2.72 | 23.85 ± 3.48 | 23.86 ± 3.43 | 0.986 | 0.02 |
| Smoking, n (%) | 18.0 (15.9) | 49.9 (16.2) | 24.4 (16.2) | 0.996 | 0.01 |
| Alcoholism, n (%) | 7.8 (6.9) | 23.5 (7.6) | 11.4 (7.6) | 0.959 | 0.03 |
| Type of atrial fibrillation: persistent, n (%) | 93.9 (82.8) | 250.8 (81.4) | 122.3 (81.3) | 0.939 | 0.038 |
| Hypertension, n (%) | 85.2 (75.2) | 224.5 (72.9) | 109.7 (73.0) | 0.912 | 0.051 |
| Diabetes, n (%) | 26.2 (23.1) | 69.4 (22.5) | 34.4 (22.9) | 0.991 | 0.015 |
| Coronary artery disease, n (%) | 27.5 (24.3) | 77.3 (25.1) | 39.8 (26.5) | 0.933 | 0.05 |
| Chronic heart failure, n (%) | 16.5 (14.5) | 50.3 (16.3) | 25.9 (17.2) | 0.863 | 0.074 |
| Myocardial infarction, n (%) | 5.2 (4.6) | 13.2 (4.3) | 6.6 (4.4) | 0.989 | 0.017 |
| Abnormal liver function, n (%) | 2.8 (2.5) | 8.2 (2.7) | 3.7 (2.5) | 0.991 | 0.014 |
| Abnormal renal function, n (%) | 11.1 (9.8) | 32.2 (10.5) | 17.0 (11.3) | 0.934 | 0.049 |

Abbreviations: LAAO：left atrial appendage occlusion; OAC: oral anticoagulation; APT: antiplatelet therapy; SD: standard deviation; BMI: body mass index; SMD: standardized mean difference.

Note: Absolute standardized mean differences were used to assess covariate balance across the three groups, and an absolute SMD <0.1 was considered to indicate acceptable balance.

**Supplementary Table S4. IPTW-adjusted Cox proportional hazards regression analyses for clinical outcomes in the external validation cohort**

| Outcome | Comparison | HR | 95% CI | *P* value |
|---|---|---|---|---|
| Composite outcome | OAC vs LAAO | 1.496 | 0.852-2.626 | 0.160 |
| | APT vs LAAO | 1.979 | 1.092-3.586 | 0.024 |
| Recurrent ischemic stroke/SE | OAC vs LAAO | 3.141 | 1.159-8.510 | 0.024 |
| | APT vs LAAO | 5.347 | 1.932-14.792 | 0.001 |
| Major bleeding | OAC vs LAAO | 1.133 | 0.315-4.072 | 0.848 |
| | APT vs LAAO | 0.896 | 0.231-3.471 | 0.874 |
| All-cause mortality | OAC vs LAAO | 1.353 | 0.657-2.787 | 0.413 |
| | APT vs LAAO | 2.391 | 1.131-5.054 | 0.022 |

Abbreviations: IPTW: inverse probability of treatment weighting; SE: systemic embolism; HR: hazard ratio; CI: confidence interval; LAAO: left atrial appendage occlusion; OAC: oral anticoagulation; APT: antiplatelet therapy.

**Supplementary Table S5. IPTW-adjusted Fine-Gray competing risk analyses for recurrent embolism and major bleeding in the external validation cohort**

| Outcome | Comparison | SHR | 95% CI | P value |
|---|---|---|---|---|
| Recurrent ischemic stroke/SE | OAC vs LAAO | 3.093 | 1.141-8.386 | 0.027 |
| | APT vs LAAO | 5.035 | 1.820-13.928 | 0.002 |
| Major bleeding | OAC vs LAAO | 1.111 | 0.312-3.961 | 0.871 |
| | APT vs LAAO | 0.839 | 0.219-3.211 | 0.797 |

Abbreviations: IPTW: inverse probability of treatment weighting; SE: systemic embolism; SHR: subdistribution hazard ratio; CI: confidence interval; LAAO: left atrial appendage occlusion; OAC: oral anticoagulation; APT: antiplatelet therapy.
